# The Voluntary Hospitals

**Published:** 1918-02-09

**Authors:** J. Courtney Buchanan, Conrad W. Thies

**Affiliations:** Honorary Secretaries, British Hospitals Association. 14 Victoria St., S.W. 1; Honorary Secretaries, British Hospitals Association. 14 Victoria St., S.W. 1


### THE EDITOR'S LETTER-BOX.

### The Voluntary Hospitals.

### Treatment of Disabled Sailors and Soldiers.

*To the Editor of* The Hospital.

Sir,—We have been asked to add to the information circulated last week, by instruction of the Council, a request that the Voluntary Hospitals in London which are willing to undertake this work will communicate direct with the County Local Committee, whose address is 43 Bloomsbury Square, W.C. 1. There are forty-five Sub-Committees in London; but the central body of the County Local Committee alone has power to conclude valid arrangements. It is hoped that this note may save the hospitals unnecessary trouble and correspondence.

We are glad of this opportunity of expressing our appreciation of the leading article which appeared in your last issue, and the sympathetic reference made therein to the arrangements made by the Council of the British Hospitals Association with the Ministry of Pensions. The whole question is one of great difficulty; but the tentative scheme has already been received with cordial approval by the hospitals generally and also by the British Medical Association.

As was indicated in the preamble to the memorandum which you were good enough to print *in extenso* last week, the meetings and resolutions of the governors and staffs of the large London hospitals who had considered this question were respectively held and passed quite independently of the meetings and resolutions held and passed by the Council of the British Hospitals Association; but it would be less than justice not to acknowledge the assistance which the resolutions of these large London hospitals have been to the Council in their careful endeavours to arrive at some common principles of action in dealing with the Ministry of Pensions.

Disabled sailors and soldiers are entitled to the best treatment that their country can provide. We believe that that treatment is to be found in, or in connection with, the voluntary hospitals; and that the governors and staffs of these institutions fully realise the occasion—with its opportunities and responsibilities.

### Increase in War Office Capitation Allowance.

We are glad to be able to report that negotiations with the War Office have been completed with the following result, viz.: There will be an increase in the capitation allowance for sick and wounded sailors and soldiers at the rate of 9d. per occupied bed per day, with 6d. per unoccupied bed per day, both dating as from October 1, 1917.

As regards the question of a differential rate for hospitals, it was agreed that it would be best to lay down a flat rate for all hospitals, but that if any hospital could show special circumstances to distinguish it from other hospitals it should make a special application to the War Office, not for any increase in the flat rate, but for a special donation. Details of the special circumstances will be instanced, *e.g.*, for fully equipped pathological department, special massage facilities, &c.

Full details of these arrangements are in preparation for forwarding to all the hospitals.—Yours faithfully,

J. Courtney Buchanan,
Conrad W. Thies,
Honorary Secretaries, British Hospitals Association.
14 Victoria St., S.W. 1, February 5, 1918.

